# Nutritional Interventions Targeting Gut Microbiota during Cancer Therapies

**DOI:** 10.3390/microorganisms9071469

**Published:** 2021-07-09

**Authors:** Emanuele Rinninella, Pauline Raoul, Marco Cintoni, Marta Palombaro, Gabriele Pulcini, Antonio Gasbarrini, Maria Cristina Mele

**Affiliations:** 1 UOC di Nutrizione Clinica, Dipartimento di Scienze Mediche e Chirurgiche, Fondazione Policlinico Universitario A. Gemelli IRCCS, Largo A. Gemelli 8, 00168 Rome, Italy; 2 Dipartimento di Medicina e Chirurgia Traslazionale, Università Cattolica Del Sacro Cuore, Largo F. Vito 1, 00168 Rome, Italy; pauline.raoul1@gmail.com (P.R.); antonio.gasbarrini@unicatt.it (A.G.); mariacristina.mele@unicatt.it (M.C.M.); 3 UOSD di Nutrizione Avanzata in Oncologia, Dipartimento di Scienze Mediche e Chirurgiche, Fondazione Policlinico Universitario A. Gemelli IRCCS, Largo A. Gemelli 8, 00168 Rome, Italy; martapalombaro@gmail.com (M.P.); gabriele.pulcini@guest.policlinicogemelli.it (G.P.); 4 Scuola di Specializzazione in Scienza dell’Alimentazione, Università di Roma Tor Vergata, Via Montpellier 1, 00133 Rome, Italy; marco.cintoni@gmail.com; 5 UOC di Medicina Interna e Gastroenterologia, Dipartimento di Scienze Mediche e Chirurgiche, Fondazione Policlinico Universitario A. Gemelli IRCCS, Largo A. Gemelli 8, 00168 Rome, Italy

**Keywords:** gut microbiota, diet, prebiotics, food, cancer treatment, toxicity, immunotherapy, chemotherapy, surgery, radiotherapy, survival

## Abstract

The gut microbiome is increasingly being recognized for its influence on intestinal and extra-intestinal disorders such as cancer. Today, diet is the most studied environmental modulator of gut microbiota, capable of altering or improving it in terms of richness and diversity. Recent evidence from several preclinical and clinical trials suggested that gut microbiota composition could modulate cancer therapies (toxicities, treatment responses) and vice versa. This review highlights the latest research on the bidirectional associations between gut microbiota and cancer. We also dissect the role of gut microbiota during cancer therapies in terms of toxicity and treatment response and, in turn, how cancer therapies could impact gut microbiota composition and functions. In this context, we summarize the state-of-the-art research regarding the role of various nutritional interventions—prebiotics, dietary strategies, and dietary restrictions—as cutting-edge possibilities to modulate gut microbiota during cancer therapies.

## 1. Introduction

The human intestine contains about 100 trillion microorganisms whose collective genome, called the “microbiome”, contains at least 100 times as many genes as the human genome does [1]. These microorganisms represent an ecosystem that continuously changes throughout life [2] and profoundly influences the physiology of their host and, in particular, the maturation of the immune system [3]. Over recent decades, numerous diseases have been associated with imbalanced gut microbiota. Indeed, patients with metabolic diseases (diabetes, obesity, hepatic steatosis), inflammatory bowel diseases, autoimmune diseases, and cancer have a gut microbiota significantly different from healthy individuals [4]. In oncology, a growing number of preclinical and clinical data have demonstrated a link between the development of cancers, as well as anti-cancer immunity, and gut microbiota [5]. Moreover, the gut microbiota appears to be involved in carcinogenesis, but at the same time, could also modulate the response and effectiveness of several anti-cancer treatments [6], making the microbiota a potential biomarker and therapeutic target for cancer treatment. On the other hand, cancer therapies may affect the function and composition of gut microbiota, and can trigger dysbiosis affecting multiple metabolic pathways, thus weakening the immune response [7]. In this context, diet is one of the key modulators of these bidirectional interactions between host-microbiota and cancer since it directly influences host homeostasis and biological processes, as well as gut microbiota composition through the microbial fermentation of nutrients [8]. Thus, the crucial mutualism between the host and gut bacteria could be altered or improved through nutritional interventions, potentially impacting intestinal barrier functions, the immune system, and the host’s response to cancer therapy. This review aims to highlight the latest research on the bidirectional associations between gut microbiota and cancer pathogenesis. We will outline the role of gut microbiota in cancer therapies in terms of toxicity and treatment response and, in turn, how cancer therapies could impact gut microbiota composition and function. In this context, we will explore the potential implications of various nutritional interventions—such as prebiotics, dietary changes, and dietary restrictions—on the modulation of gut microbiota during cancer therapies.

## 2. Gut Microbiota and Cancer Pathogenesis

A high diversity and richness of gut microbiota characterize intestinal eubiosis which is associated with health status; on the other hand, growing evidence reports that gut microbiota dysbiosis is closely associated with carcinogenesis. Indeed, altered gut microbiota composition and function are involved in tumorigenesis and/or tumor growth, such as in the development of colorectal cancer (CRC) [9] and pancreatic [10], liver [11], and breast [12] cancers. 

Several studies demonstrated that the risk of breast cancer (BC) development could be associated with the composition, as well as thefunction, of gut microbiota. One study compared fecal samples of women with grade 1 BC to those with grade 3 BC [13]. The abundance of *Blautia* spp. increased in women with grade 3 BC. Moreover, quantities of *Bifidobacterium* spp. and *Faecalibacterium prausnitzii* significantly differed between clinical stages [13]. Compared with healthy subjects, another study found a lower gut microbial diversity and particularly increased levels of *Clostridiaceae*, *Faecalibacterium*, and *Ruminococcaceae* in fecal samples of postmenopausal BC women [14]. Alterations to gut bacterial composition could impact the function of the microbiota metabolites involved in breast cancer carcinogenesis. In BC, a recent animal study supported this hypothesis by assessing the association between risk of BC development, gut microbiota, and cadaverine biosynthesis [15]. Cadaverine is a metabolite produced by the decarboxylation of lysine by lysine decarboxylase enzymes. These specific enzymes are mainly expressed by gut bacterial species. In fecal deoxyribonucleic acid (DNA) samples of early-stage BC women, compared with those of controls, Kovàcs et al. reported a lower abundance of proteins and genes involved in cadaverine production and lower protein expression of *E. coli*, which is associated with the production of lysine decarboxylase enzymes [15]. Thus, cadaverine production could be one of the regulators of early BC through gut microbial pathways. 

Intestinal dysbiosis and increased bacterial translocation also contribute to the pathophysiology of chronic liver disease and hepatocarcinogenesis [16]. Dysbiosis and leaky gut could promote the progression of cirrhosis and the development of hepatocellular carcinoma (HCC) via multiple mechanisms. Unbalanced microbiota could stimulate the production of cancer-promoting metabolites such as deoxycholic acid and the production of lipopolysaccharide (LPS). Increased production of LPS could promote hepatic inflammation, fibrosis, and proliferation and activate anti-apoptotic signals [16]. Interestingly, in mice, targeting the gut-microbiota–liver axis could directly impact the development of HCC [17,18]. Indeed, a model of rat hepatocarcinogenesis was treated with penicillin or dextran sulfate sodium (DSS) to disrupt the intestinal homeostasis and, successively, gut homeostasis was restored by treatment with probiotics [18]. This study reported a significantly lower abundance of *Lactobacillus* spp., *Bifidobacterium* spp., and *Enterococcus* spp. and an intestinal inflammation induced by penicillin or DSS. Inversely, the administration of probiotics counteracted intestinal inflammation and decreased liver tumor growth.

As regards CRC, a recent meta-analysis [9] of fecal metagenome studies [17,18,19] revealed specific gut microbial signatures. Indeed, in fecal samples of CRC patients, various bacterial abundances were reported to be higher compared with healthy controls, such as *Bacteroides fragilis*, *Escherichia coli*, *Streptococcus gallolyticus*, *Enterococcus faecalis*, and *Fusobacterium nucleatum*, while some bacterial species were lower in abundances such as *Bacteroides vulgatus*, *Clostridium coccoides*, and *Faecalibacterium prausnitzii*. Furthermore, mucin catabolism genes and depleted carbohydrate degradation genes have been detected in the functional analysis of CRC metagenomes [9]. Preclinical models of gavage of stool from CRC patients revealed induction of polyp formation and an alteration of the immune system in conventional and germ-free mice fed with stool, compared with controls [19]. Specifically, Purcell et al. showed a positive association between high levels of *Bacteroides fragilis* and an increased number of early-stage carcinogenic lesions [20]. The *Bacteroides fragilis* toxin gene may be prevalent in the mucosal colon tissue [21]. *Bacteroides fragilis* produces the *Bacteroides fragilis* toxin which is associated with the presence of acute diarrhea and inflammatory bowel disease [22]. *Fusobacterium nucleatum* also represents one of the main gut bacteria involved in the development and progression of CRC as it was detected in primary colon adenoma and CRC [23]. Indeed, recent studies reported a high abundance of *Fusobacterium* in CRC subjects compared with healthy subjects [24]. Moreover, there are significant correlations between local tumor necrosis factor-alpha (TNF-α) and *Fusobacterium*, as well as *Fusobacterium* and interleukin (IL)-10. These results support the hypothesis that there is a link between the abundance of *Fusobacterium* in colonic mucosa and adenomas and mucosal inflammation [21]. Furthermore, Yu et al. defined potential differential bacterium patterns between recurrent and non-recurrent CRC patients [25]. In recurrent CRC tissues, compared with non-recurrent CRC tissues, they found higher abundances of *Fusobacterium*, *Anaerosporobacter*, *Parvimonas*, *Peptostreptococcus*, and *Prevotella*. Specifically, *Fusobacterium nucleatum* was the most enriched bacterium in patients with recurrent CRC, suggesting that *Fusobacterium nucleatum* may also play a role in CRC recurrence [25]. As regards gastric cancer (GC), *Helicobacter pylori* is known to induce gastric inflammation and GC [26]. Additionally, *Clostridium*, *Fusobacterium*, and *Lactobacillus* spp. were frequently abundant in patients with GC compared with controls, demonstrating the presence of a GC-specific gut bacterial signature [27]. 

All these examples demonstrate that pathogenic gut microbial signatures are involved in a wide range of cancers through mucosal intestinal inflammation and modulation of the host’s immune system. However, it remains unclear whether gut microbial variations—in terms of composition and function—might cause cancer pathogenesis or whether, inversely, the tumor microenvironment could modify the gut microbial composition. Interestingly though, recent studies reported tight associations between gut microbiota and cancer therapies and their outcomes. 

## 3. Gut Microbiota and Cancer Therapy: Bidirectional Interactions

Cancer therapies including chemotherapy, immunotherapy, and radiology may trigger side effects, some of which can impact the survival outcomes of oncological patients. Surgery may also cause postoperative complications impacting survival. Here, we highlight the emerging role of gut microbiota in modulating the response, efficacy, and toxicity of different cancer therapies and, in turn, how such therapies could impact gut microbiota. 

### 3.1. Potential Roles of Gut Microbiota in the Modulation of Response to Cancer Therapy 

Several studies have recently shown that gut microbiota could influence responses to therapies and drugs through different mechanisms. 

#### 3.1.1. Chemotherapy

García-González et al. used the model of *Caenorhabditis elegans* and its bacterial diet (enriched with either *E. coli* or *C. aquatica* ) to study how bacteria affect the *C. elegans* response to chemotherapeutics such as 5-fluorouracil, 5-fluoro-2′-deoxyuridine, and camptothecin [28]. They demonstrated that the gut bacteria *E. coli* and *C. aquatica* affect the response to camptothecin and 5-fluoro-2′-deoxyuridine. Even though this study focused on the simplest organism system with only two bacterial species, these findings highlight the possible impact of gut bacteria on the efficacy of some chemotherapeutics used to treat CRC in humans. 

In mice models, cyclophosphamide is one of the most studied chemotherapy drugs. Cyclophosphamide belongs to the class of alkylating agents and is used to treat various forms of cancers. In a mouse model study, Viaud et al. demonstrated, in tumor-bearing mice treated with antibiotics (specific pathogen-free) or germ-free, a reduction in T helper 17 cells [29] compared with tumor-bearing control mice. Moreover, the tumors of the antibiotic-treated or germ-free mice were resistant to cyclophosphamide [29]. These results suggest that gut bacteria could stimulate the production of a specific subset of “pathogenic” T helper 17 cells, improving response to cyclophosphamide treatment. Interestingly, another mouse model reported that the presence of the gut bacteria *Alistipes* and *Ruminococcus* could be positively linked with the capacity of tumor-associated myeloid cells to secrete TNF-α, thereby enhancing the anti-cancer effect [30]. Moreover, Daillère et al. identified two bacterial species, *Enterococcus hirae* and *Barnesiella intestinihominis*, involved in the response to cyclophosphamide therapy [31]. *Enterococcus hirae* could translocate from the small intestine to secondary lymphoid organs and increase the intratumoral CD8/Treg ratio, while *Barnesiella intestinihominis* could promote the infiltration of interferon-gamma (IFN-γ)-producing γδT cells in cancer lesions [31]. Heshiki et al. recently attempted to find a microbiota signature, independent of cancer type and heterogeneity, using a combination of human feces shotgun metagenomic sequencing in both in vitro and in vivo mouse models [32]. Increased abundance of *Bacteroides ovatus* and *Bacteroides xylanisolvens* could be associated with several treatment outcomes. Specifically, in a murine lung cancer model, oral gavage of these specific bacteria significantly increased the efficacy of erlotinib and induced the expression of IFN-γ [32]. Finally, a recent study [33] went further by investigating the effect of gut microbial metabolites on the efficacy of oxaliplatin—a chemotherapy drug used to treat colon or rectal cancer in combination with fluorouracil and leucovorin. The authors demonstrated that butyrate treatment stimulates the antitumor cytotoxic CD8+ T cell response both in vitro and in vivo. Moreover, in humans, cancer patient responding to oxaliplatin reported higher serum butyrate levels than non-responding patients, which could positively impact the regulation of CD8+ T cell immunity and facilitate chemotherapy efficacy [33]. Thus, “immunostimulatory” gut bacteria and their related metabolites could be targeted to alleviate the deleterious effect of microbiota depletion in mice or even optimize responses to anti-cancer drugs [30]. However, it is advisable to take caution before translating these observations into humans. Antibiotic use in humans rarely leads to a complete depletion of the gut microbiota, and dysbiosis is usually transient [34]. Moreover, the aforementioned findings suggest inflammatory cytokines enhance the efficacy of cancer therapy, however, some controversial evidence indicates that elevated production of IL-17, in response to translocating commensal bacteria, could promote the progression of CRC [35]. 

#### 3.1.2. Immunotherapy

Immune checkpoint inhibitors (ICIs) represent a potential revolution in the field of cancer therapy. Immune checkpoints are inhibitory molecules, expressed on the surfaces of lymphocytes (B cells, T cells, and natural killer cells), mediating immunotolerance as a normal function of the immune system. The most studied ICIs are programmed cell death protein 1 (PD-1), together with its ligand PD-L1—expressed by both antigen-presenting cells (APCs) and cancer cells—and cytotoxic T lymphocyte antigen-4 (CTLA-4) [36]. Cancer cells can activate different immune checkpoint pathways leading to stronger immune responses. On the other hand, when these checkpoints are blocked, T cells can kill cancer cells without inhibition. Treating cancer patients with antibodies to inhibit immune checkpoint proteins represents the earliest immunotherapeutic cancer therapy. Blocking the binding of PD-L1 to PD-1 with an immune checkpoint inhibitor allows the T cells to kill tumor cells [37]. Growing evidence demonstrates the impact of gut microbiota on ICI efficacy. Routy et al. demonstrated, in non-small-cell lung cancer (NSCLC) patients receiving PD-1/PD-L1 monoclonal antibody treatment, a significant enrichment of *Akkermansia muciniphila* in the stools of responders compared with non-responders [38]. In melanoma patients, two other studies also reported that gut microbial composition influences PD-1 immunotherapy response with an increase in *Bifidobacterium longum*, *Collinsella aerofaciens*, *Enterococcus faecium,* and *Ruminoccocus* spp. associated with better immunotherapy responses [5,39]. In mice and patients with melanoma following anti-CTLA-4 therapy, T cell responses specific for *Bacteroides thetaiotaomicron* or *Bacteroides fragilis* were associated with the efficacy of CTLA-4 blockade [40]. Moreover, microbial metabolites such as short-chain fatty acids (SCFAs), especially butyrate, are associated with anti-PD-1 and anti-CTLA-4 therapies [41,42]. In terms of toxicity, gut microbiota, especially Bacteroidetes phylum, could be negatively associated with the development of checkpoint-blockade-induced colitis [43,44]. Specifically, this phylum was associated with a lower risk of developing colitis in melanoma patients treated with anti-CTLA-4 therapy [43]. 

#### 3.1.3. Radiotherapy

The influence of gut microbiota on radiation therapy in terms of responses has also been investigated. Radiation therapy exerts its genotoxic effect directly on cancer cells [6]. At the same time, radiotherapy activates both immune-stimulating and immunosuppressive responses. When this balance is shifted towards a pro-inflammatory response, with the activation of antigen-presenting dendritic cells and upregulation of CD4 Th1 and CD8 cytotoxic T cells, anti-cancer activity can also be seen distant from the radiation field [45]. Indeed, a mouse model described the impact of gut microbiota composition on radiation therapy delivered to sites distal to the gut and demonstrated that the gut microbiota can modulate dendritic cell antigen presentation to improve radiation therapy-mediated antitumor responses [46]. Interestingly, fecal microbiota transplantation increased the survival rate of irradiated animals, elevated peripheral white blood cell counts, and alleviated gastrointestinal toxicities and intestinal epithelial integrity in irradiated mice [47]. The effects of radiation therapy are lessened in terms of endothelial cell apoptosis and induction of lymphocyte infiltration in germ-free mice than in conventional mice [48]. Hence, we can argue that gut microbiota could regulate the response and attenuate the toxicity of radiation therapy, repairing irradiation-induced damage. Interestingly, in women with gynecological cancer, a recent systematic review of cohort studies [49] highlighted that patients who developed diarrhea as an adverse effect of radiotherapy had, before treatment, an increased abundance of *Bacteroides*, *Dialister*, and *Veillonella*, and a decreased abundance of *Clostridium XI* and *XVIII*, *Faecalibacterium*, *Oscillibacter*, *Parabacteroides,* and Prevotella, compared with those who did not develop diarrhea.

#### 3.1.4. Surgery

Complications following gastrointestinal surgery in CRC patients could be also modulated by gut microbiota [50]. Evidence suggests that *Lactobacillus* spp. and *Akkermansia muciniphila* could regulate the intestinal wound healing process via reactive oxygen species-dependent (ROS) mechanisms [50]. A mouse model study also demonstrated that the colonization of germ-free mice with *B. thetaiotaomicron* could positively influence nutrient absorption, mucosal barrier integrity, and angiogenesis [51]. Inversely, pathogenic bacteria such as *Serratia marcescens* could promote the development of surgical complications. Indeed, a collagenolytic strain of *Serratia marcescens* is capable of causing anastomotic leakage in mice [52]. Thus, future studies should deeply analyze gut microbiota to identify the potential effect of the gut microbes on the processes of wound healing and anastomotic leakage reduction. 

We can conclude that clinical outcomes of cancer treatments could be substantially modulated by the abundances of specific host gut bacteria, representing key activators of the immune system. In the future, we imagine that the identification of the gut microbial signature of a cancer patient could help clinicians to predict the clinical outcomes of cancer therapies, critical for disease-monitoring and treatment decision-making. However, we have to consider that cancer therapies could further perturb the immune response triggered by dysbiosis, as we try to show in the next paragraph. 

### 3.2. Cancer Therapies Impact Gut Microbiota Composition and Functions

#### 3.2.1. Chemotherapy 

Irinotecan is a key anti-cancer drug for the treatment of metastatic CRC; one of the most important toxic effects is severe diarrhea [53]. One study showed that irinotecan chemotherapy alters intestinal microbiota in tumor-bearing rats, increasing the abundance of *Clostridium clusters I*, *XI*, and Enterobacteriaceae, particularly after dose-intensive therapy [54]. 5-Fluorouracil (5-FU), used in the treatment of numerous cancers (such as colorectal, breast, and liver), is an anti-metabolite acting as a pyrimidine antagonist which often triggers side effects such as diarrhea [53]. An observational study investigated the effects of 5-FU, epirubicin, and cyclophosphamide on the intestinal barrier function and gut peptides in breast cancer patients [55] reporting alterations in intestinal permeability associated with modifications to the levels of glucagon-like peptide-2, ghrelin, and epidermal growth factor. In patients experiencing diarrhea induced by treatment, increased intestinal permeability was found in comparison with patients without diarrhea. Additionally, Van Vliet et al. [56] demonstrated that chemotherapy treatment in pediatric patients with acute myeloid leukemia receiving anti-microbial prophylaxis leads to a decrease in anaerobic bacteria and an increase in potentially pathogenic aerobic enterococci, suggesting that these gut microbiota disturbances will further increase the risk of gram-positive aerobic infections. Another study investigated the association of gut microbiota variations with paclitaxel-induced neuropathic pain in C57BL/6 (B6) and 129SvEv (129) mice, respectively sensitive and resistant to chemotherapy [57]. Paclitaxel is a frontline chemotherapeutic drug that often causes chemotherapy-induced peripheral neuropathy [57]. In the paclitaxel-induced analyses of the study, a decrease in the relative abundance of *Akkermansia muciniphila* in the B6 microbiota from paclitaxel day 0 to paclitaxel day 10 suggested that *Akkermansia muciniphila* could inhibit pain, by promoting gut barrier function [58]. Consequently, paclitaxel chemotherapy may cause barrier dysfunction resulting in increased systemic exposure to bacterial products and metabolites, thereby promoting systemic inflammation leading to pain sensitivity through a reduction in the abundance of *Akkermansia muciniphila* [57]. 

#### 3.2.2. Immunotherapy

Given that gut microbiota is closely associated with the immune system, immunotherapies also impact gut microbiota composition and function. In HCC patients receiving anti-PD1 therapy, compositional microbial variations occurred after week 3 with an increase in *Escherichia coli* in non-responder stools and an enrichment of *Lactobacillus*, *Ruminococcaceae,* and *Akkermansia muciniphila* in responder stool samples [59]. Another study found a decrease in *Bacteroidales* and *Burkholderiales* abundances and an increase in Clostridiales in patients with melanoma receiving anti-CTLA4 therapies [40]. However, despite specific variations in bacterial abundances, few studies reported a relatively stable microbial composition during immunotherapy [44,60].

#### 3.2.3. Surgery

Recently, outstanding advances have been made in the surgery of cancer patients, and the impact of surgery on gut microbiota is being increasingly studied, notably in gastrointestinal cancer patients. 

A recent study investigated the effect of gastrectomy for GC on the gut microbiome and metabolome and its association with post-gastrectomy outcomes by comparing fecal samples from patients with a history of gastrectomy with those from healthy controls [61]. The gut microbiota in the gastrectomy group showed significant variations in gut microbial species diversity and richness, which can be linked to the reconstruction of the gastrointestinal tract of patients with GC. These compositional microbial variations could lead to gastrectomy-associated alterations in microbial functions, such as nutrient transport and biosynthesis of organic compounds, which might relate to changes in post-gastrectomy metabolism [61]. A Chinese study analyzed fecal microbiota shifts of patients with GC before and after radical distal gastrectomy during their hospital stays [62]. Surgery intervention had significant effects on the gut microbial composition when comparing the postoperative group with the preoperative group. The relative abundances of *Akkermansia muciniphila, Escherichia/Shigella*, *Lactobacillus*, and *Dialister* significantly varied in the perioperative period. Furthermore, an increased abundance of *Escherichia/Shigella*, *Veillonella*, and *Clostridium XVIII* and a decreased level of *Bacteroides* were reported in the gut microbiota of patients who underwent gastrectomy compared with healthy controls [62]. These microbial changes are probably due to surgical stress and other perioperative factors. 

As regards CRC surgery, recent animal studies have revealed significant alterations in the composition of the gut microbiota after colorectal resection, with a lower abundance of Bacteroidetes and Proteobacteria such as *Enterobacteriaceae* and *Rhodospirillaceae* [63]. Moreover, this surgery-associated microbial dysbiosis was associated with increased IL10 and IL12 gene expression and decreased gene expression of TNF following surgery [63]. In CRC patients, a growing number of recently published studies demonstrated surgery-associated reshaping of the fecal microbiota for post-surgery CRC patients, compared with healthy controls and pre-surgery CRC patients. At species levels, the significant postoperative microbial changes were a decreased abundance in *Bacteroides* [64,65], *Bifidobacterium* [64,65], *Clostridium* [64], *Prevotella* [64,65], *Klebsiella* [66], *Faecalibacterium* [65], and *Parabacteroides* [65], and an increased abundance in *Enterococcus* [64], *Pseudomonas* [64], *Staphylococcus* [65], *Escherichia-Shigella* [65], *Enterobacteriaceae* [65], and *Streptococcus* [64]. However, another study reported a reduction in *Escherichia-Shigella* and an increase in *Enterococcus* and *Parabacteroides* [67]. At the phylum level, a decreased number of Firmicutes and Bacteroidetes [68], with a reduction of Firmicutes/Bacteroides ratio and an increased number of Proteobacteria [66,68] were reported. All these alterations could be due to perioperative antibiotic administration possibly altering intestinal microbiota and resulting in impaired host immunity or metabolism [69]. Consequently, the disruption of this delicate gut barrier homeostasis associated with surgery stress could promote adverse inflammatory outcomes in these patients.

#### 3.2.4. Radiotherapy

Radiotherapy leads to anti-tumor responses through the immune response [70]; consequently, and similarly to other cancer therapies, gut microbial compositional variations occur during this type of treatment. An animal study reported luminal and mucosa-associated dysbiosis in irradiated mice compared with control mice at two post-radiation time points and correlated it with an increase in TNF-α, IL-1β, and IL-6 expression [71]. Another recent mouse study found an increase in the level of the genera *Alistipes* and a decrease in that of *Prevotella* in the large intestine after radiation exposure [72]. In humans, Mitra et al. [73] showed a decreased gut microbiome diversity in cervical cancer patients following chemoradiation therapy [73], while in another study of gynecological cancer patients who received pelvic radiotherapy, levels of the phyla Firmicutes decreased and those of *Fusobacterium* increased [74]. Although these results need to be confirmed, we can hypothesize that radiation-induced gut microbiota changes could be associated with changes in the gut microbiota leading to alteration of the intestinal mucosa and consequently gut inflammation [71].

All these findings pave the way to new strategies to modulate gut microbiota and improve oncological treatment outcomes. One such strategy is diet, a well-known modulator of gut microbiota composition and function [75]. Hence, the number of studies regarding the effect of nutritional interventions during cancer therapy on gut microbiota and clinical outcomes is growing. Subsequently, we dissect the potential role of several nutritional interventions as gut microbiota modulators in improving cancer therapy responses in cancer patients. 

## 4. Nutritional Interventions Modulating Gut Microbiota during Cancer Therapy

### 4.1. Prebiotics

Prebiotics are fibers promoting the growth of specific groups of anaerobic colonic indigenous bacteria; they include inulin, fructo-oligosaccharide (FOS), and galactooligosaccharides (GOS). These carbohydrates are non-digestible by endogenous enzymes in the small intestine, but are actively fermented by the colonic bacteria, selectively promoting the growth of beneficial bacteria such as *Bifidobacterium* spp. [76]. Increased levels of *Bifidobacterium* spp. have been reported to reduce tumor incidence and/or growth [77]. Moreover, inulin and oligofructose—a subgroup of inulin consisting of polymers with a degree of polymerization ≤10—could reduce the incidence of aberrant crypt foci in the colon of rats previously injected with a chemical carcinogen [78].

Taper et al. studied the effect of 15% inulin or oligo-fructose incorporated into the basal diet of experimental animals on chemotherapy responses [79,80,81]. Both inulin and oligofructose have been shown to potentiate the therapeutic effects of all six cytotoxic drugs (5-FU, doxorubicin, vincristine, cyclophosphamide, methotrexate, and cytarabine) that are representative of the different groups of cytotoxic drugs classically used in human cancer treatment [80]. These results confirm those of a previous study of the same group, in which a significant booster effect by inulin was observed for cyclophosphamide (the response increased by 47%) [81]. On the other side, no negative result of the adjuvant therapy induced by inulin or oligofructose was observed [81].

A randomized, double-blind, placebo-controlled trial studied the effect of a mixture of fiber (50% inulin and 50% FOS) on microbiota in gynecological cancer patients undergoing radiotherapy [82]. The group consuming the prebiotic mixture experienced a faster recovery of *Lactobacillus* spp. and *Bifidobacterium* spp. counts 2 weeks after completion of radiotherapy, compared with the placebo group [82], leading to an improvement in the consistency of stools [83]. Diarrhea is also a common complication of enteral nutrition, which affects recovery and prolongs the length of hospital stay (LOHS), especially in postoperative patients with GC. A study of 120 patients with GC in three groups (fiber-free nutrition formula, fiber-enriched nutrition formula, and fiber- and probiotic-enriched nutrition formula) showed that LOHS in both fiber groups was shorter than that in the fiber-free group, with a reduction of diarrhea symptoms [84]. A phase II randomized controlled trial of patients with localized anal canal squamous cell cancer was recently initiated to investigate the effect of the gut microbiota–prebiotics associations during radiotherapy on treatment effectiveness and clinical outcomes [85]. 

A randomized, double-blind clinical trial investigated the effects of prebiotics (FOS, xylooligosaccharides, polydextrose, and resistant dextrin) on gut microbiota and immune function in 140 perioperative patients with CRC [86]. Prebiotic consumption led to an increase in the abundance of *Bifidobacterium* and *Enterococcus* and a reduction in the abundance of *Bacteroides* levels in the preoperative period, compared with placebo [86]. In the postoperative period, the abundance of *Enterococcus*, *Bacillus*, *Lactococcus*, and *Streptococcus* increased in the control group, compared with the prebiotic group. Moreover, the abundance of harmless strains of *Escherichia-Shigella* increased after prebiotic intake in the postoperative period. Notably, the richness of intestinal microbiota from preoperative to postoperative decreased in the non-prebiotic group. As regards immunological markers, prebiotic intake produced significant effects on immunologic indices in both the preoperative and postoperative periods [86]. Prebiotics significantly increased serum levels of immunoglobulin (Ig)G, IgM, and transferrin in the preoperative period, and levels of IgG, IgA, suppressor/cytotoxic T cells (CD3+CD8+), and total B lymphocytes in the postoperative period, compared with controls [86]. 

Ruault et al. [87] investigated variations in biological markers before and after 3 months of daily intake of 10 g of FOS in 74 French patients (26 subjects with small colorectal adenomas, 18 with large adenomas, and 30 with no adenoma). Butyrate was significantly increased in the adenoma groups after 3 months of daily intake of prebiotics, compared with baseline. Cholic acid, chenodeoxycholic acid, total primary bile acids, and ursodeoxycholic acid increased and fecal lithocholic acid decreased in subjects without adenoma, suggesting a significant role of FOS intake in modulating the colonic microenvironment [87]. 

Inulin has been reported to induce *Bifidobacterium* spp. and *Akkermansia muciniphila* abundance [88,89]. An in vitro study showed that the cultivation of fecal samples with inulin and mucin increased the relative abundance of gut microbial species implicated in tumor growth control in Rnf5−/− mice [90]. A recent study went further, demonstrating that inulin consumption leads to an enrichment of bacterial taxa that promotes anti-tumor immunity [89]. Indeed, the addition of the prebiotics inulin or mucin to the diet of C57BL/6 mice could induce anti-tumor immune responses. Interestingly, in the same study, mucin failed to inhibit tumor growth in germ-free mice, indicating that the gut microbiota is required for the activation of the anti-tumor immune response [90]. Inulin could limit tumor growth in mouse models of colon cancer and NRAS mutant melanoma. It also enhanced the efficacy of a MEK inhibitor against melanoma affecting the MAPK/ERK pathway, which is overactive in melanoma [90]. 

Thus, dietary treatment with inulin or oligofructose could potentiate the effects of cancer chemotherapy through modulation of gut microbial composition and the immune system. However, the number of studies is currently low and larger studies are required to confirm these findings [91]. 

### 4.2. Fermented Foods

Probiotics are the living organisms in our gut that contribute to healthy status [92]. They can be found in fermented foods such as yogurt, kefir, sauerkraut, and kimchi [93]. Among fermented dairy foods, natural yogurt, sweetened yogurt, and matured cheese were the most consumed [94]. Gonzalez et al. analyzed the relationship between the intake of fermented dairy foods within the regular diet, the gut microbial profile, and health-related biomarkers in 130 healthy adults [94]. Natural yogurt consumers showed increased fecal levels of *Akkermansia* than non-consumers, and sweetened yogurt intake was associated with lower levels of *Bacteroides* [94].

An interesting randomized controlled trial assessed whether a *Bifidobacterium*-containing yogurt product could modulate the gut microbiome and clinical outcomes of metastatic renal cell carcinoma (mRCC) patients initiating vascular endothelial growth factor-tyrosine kinase inhibitors (VEGF-TKIs) [95]. They randomized patients into the probiotic-supplemented group (receiving two servings of 120g probiotic yogurt product daily) or the probiotic-restricted control group. Probiotic supplementation successfully increased the abundance of *Bifidobacterium* spp. Additionally, in patients with clinical benefit (i.e., response of either complete/partial response or stable disease for over 6 months), *Barnesiella intestinihominis* and *Akkermansia muciniphila* were significantly more abundant compared with patients with no clinical benefit. [95]. This is the first study prospectively assessing the impact of fermented food on clinical outcomes in cancer patients undergoing chemotherapy. Furthermore, a recent Korean study showed that a polysaccharide called BF-E2-P, isolated from fermented barley, could activate the innate immune system and anti-tumor metastasis in mice [96]. BF-E2-P stimulated macrophages and cytokine production. Also, intravenous administration of BF-E2-P increased natural killer cell-mediated cytotoxicity against cancer cells and increased the production of IFN- γ [96], counteracting tumor growth [97]. Future randomized controlled studies are required to confirm these results and better understand the potential role of fermented functional food supplementation as a therapeutic tool. 

### 4.3. Ketogenic Diet (KD)

KD represents a therapeutic dietary treatment for epilepsy and GLUT1 deficiency syndrome [98]. KD is an isocaloric high-fat diet resulting in a reduction in carbohydrate intake [8]. KD is specifically designed to trigger ketosis and is emerging as a therapy for cancer patients [99]. Van der Heiden et al. reported that tumor cells take up higher amounts of glucose than the surrounding tissue and are capable of producing lactate through the aerobic glycolytic pathway [100]. Therefore, limiting glucose availability to cancer cells could deprive them of energy production, and consequently decrease tumor proliferation. Recently, in vitro and in vivo mice models have demonstrated that KD inhibits glycolysis and cancer cell proliferation, leading to anti-tumor effects in patients [101,102]. However, the specific role of the microbiota in mediating anti-tumor effects induced by KD during cancer therapy remains unknown. This potential role of gut microbiota has been studied in other diseases such as autism or epilepsy [103,104]. Indeed, in mice affected by autism spectrum disorder, KD could normalize excessively high levels of *Akkermansia muciniphila*, significantly increasing the Firmicutes/Bacteroidetes ratio [103]. In infants with refractory epilepsy, KD decreases the frequency of seizures, increases the abundance of *Bacteroides* and *Prevotella*, and decreases that of *Cronobacter* [104]. In cancer patients undergoing cancer therapies, such beneficial changes can be imagined but remain to be tested. 

### 4.4. Dietary Restrictions

Dietary restrictions include: (i) caloric restriction (CR) (defined by a 20–50% reduction in energy without malnutrition or reduction in essential nutrients occurring), but also (ii) time-restricted feeding (TRF) (which provides food intake in a 4- to 12-h time window), (iii) intermittent fasting (IF) (which provides an alternation of 24-h fasting with a 24-h ad libitum eating period), or (iv) fasting-mimicking diet (FMD) (a reduction of caloric intake for five consecutive days, through a low-caloric vegetable-based diet, before returning to normal eating cycles, once a month) [105]. In recent decades, there has been growing interest in dietary restrictions for their role in delaying the onset and burden of cancers, as well as many other non-communicable diseases [106,107]. Indeed, Lee et al. demonstrated that cycles of starvation were as effective as chemotherapeutic agents in delaying cancer progression and increased the effectiveness of these drugs against melanoma, glioma, and breast cancer cells by increasing oxidative stress, caspase-3 cleavage, DNA damage, and apoptosis [108]. Other in vitro and in vivo studies confirmed these results by assessing the positive effect of short-term fasting on the efficacy of chemotherapy [109,110] and radiotherapy [111]. One of the main drivers through which dietary restrictions influence metabolic improvement and the immune system could be the gut microbiota. Indeed, we have previously highlighted that the gut microbiota is involved in cancer development and therapy response by maintaining or disrupting gut homeostasis and consequently impacting the gut barrier and immune system. Thus, dietary restrictions may counteract gut dysbiosis, thereby positively influencing host metabolism and the immune system; precisely, dysregulation of gut permeability and bacterial translocation of luminal contents to the underlying mucosa impact the immune system. Intestinal immune homeostasis is regulated by the crosstalk between epithelial cells and dendritic cells [112]. Thus, the modulation of microbiota through these dietary changes could potentiate treatment outcomes in cancer patients. However, until now, reliable data on this topic are lacking. On the other hand, it is important to consider that dietary restrictions such as fasting can worsen cachexia syndrome, which affects about half of all cancer patients [113]. Future research should determine the optimal therapeutic windows of these nutritional interventions based on the gut microbiota composition, the cancer type, and the characteristics of the patient. To our best knowledge, no clinical studies have evaluated the effect of gut microbiota modulation through dietary restrictions on clinical outcomes of cancer therapies.

## 5. Conclusions and Perspectives

**Figure 1:**
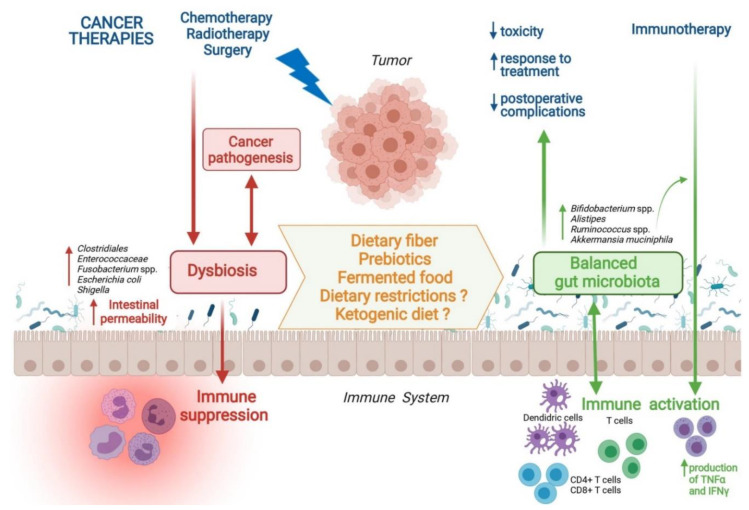
Nutritional Interventions Modulating Gut Microbiota and Clinical Outcomes during Cancer Therapy. Abbreviations: spp., species; TNFα, tumor necrosis factor-alpha; IFNγ, interferon-gamma.

Modulating gut microbiota through nutritional interventions is promising (Figure 1), even if there is a huge lack of data regarding the impact of nutritional supports on the composition of gut microbiota and their associations with clinical cancer therapy outcomes. We can only hypothesize that a highly rich and diverse microbiota could improve oncological results. The enrichment of gut microbiota with beneficial bacteria such as *Bifidobacterium* or *Akkermansia* could be potentiated by prebiotic intake and fermented food consumption. KD, caloric restriction, and fasting are interesting approaches to explore but, to date, they cannot be recommended considering the high prevalence of malnutrition in cancer patients. Large studies are required to assess whether a multidisciplinary oncological team, including nutritionists, could be able to provide cancer patients with detailed and personalized dietary guidelines to improve gut homeostasis and achieve better treatment outcomes. Thus, future large studies are needed to understand how to fully harness the gut microbiota as a potential therapeutic tool through personalized nutritional interventions.

## Data Availability

Not applicable.
